# Research, recruitment and observational data collection in care homes: lessons from the PACE study

**DOI:** 10.1186/s13104-019-4543-2

**Published:** 2019-08-14

**Authors:** Danni Collingridge Moore, Sheila Payne, Lieve Van den Block, Maud ten Koppel, Katarzyna Szczerbińska, Katherine Froggatt, PACE (Palliative Care for Older People in care and nursing homes in Europe) Consortium, Lieve Van den Block, Borja Arrue, Ilona Baranska, Danni Collingridge Moore, Luc Deliens, Yvonne Engels, Harriet Finne-Soveri, Katherine Froggatt, Giovanni Gambassi, Viola Kijowska, Maud ten Koppel, Marika Kylanen, Federica Mammarella, Tinne Smets, Bregje Onwuteaka-Philipsen, Mariska Oosterveld-Vlug, Roeline Pasman, Sheila Payne, Ruth Piers, Lara Pivodic, Jenny van der Steen, Katarzyna Szczerbińska, Nele Van Den Noortgate, Hein van Hout, Anne Wichmann, Myrra Vernooij-Dassen

**Affiliations:** 1 International Observatory on End of Life Care, Lancaster University, Lancaster, UK; 2 VUB-UGhent End of Life Care Research Group, Vrije Universiteit Brussel (VUB), Brussels, Belgium; 3 Expertise Center for Palliative Care, Department of Public and Occupational Health, Amsterdam UMC, Vrije Universiteit Amsterdam, Amsterdam Public Health Research Institute, Amsterdam, The Netherlands; 4 Unit for Research on Aging Society, Department of Medical Sociology, Chair of Epidemiology and Preventive Medicine, Faculty of Medicine, Jagiellonian University Medical College, Krakow, Poland

**Keywords:** Care home, Nursing home, Long term care facility, Palliative care, Observational study, Epidemiology

## Abstract

**Objective:**

Care homes are a common place of death for older adults, especially those with complex health needs or dementia. Representative, internationally comparable data on care home facilities and their residents is needed to monitor health and wellbeing in this population. Identification and collection of data from care homes can be challenging and often underreported. This paper draws on the experiences of the PACE study, a cross sectional mortality follow back study conducted in six European countries.

**Results:**

Multiple challenges were encountered in creating a sampling framework and contacting, recruiting and retaining care homes in the PACE study. Recruiting a randomly identified, representative cohort from a stratified sampling framework was problematic, as was engaging with care homes to ensure high response rates. Variation in the funding of care homes across the six countries involved in the study may explain the additional challenges encountered in England. Awareness of the challenges encountered in England in implementing an international study in care homes can inform the design and implementation of future studies within care homes. Further discussion is needed to determine the barriers and facilitators to conducting research in care homes, and how this is shaped by the focus of the study.

## Introduction

Long term care facilities, or care homes, are becoming a common place of death for older adults [1, 2]. Ensuring that appropriate services are available to meet the health needs of this population will require accurate, good quality data. Research in this area is increasingly complex; in addition to the challenges of conducting research with older adults [3], the difficulties in obtaining ethical approval, accessing care home residents through gatekeepers, gaining informed consent and collecting data from residents have been explored [4–8]. The experiences of involving care homes as facilities in research, rather than residents, is less understood.

The Palliative Care for Older People in care and nursing homes in Europe (PACE) programme of research, centred on improving palliative care in long-term care facilities across Europe [9]. This paper reflects on the experience of setting up and running a cross sectional study of resident deaths within care homes, conducted in six European countries: United Kingdom (England), the Netherlands, Belgium, Finland, Poland and Italy [10]. The study aimed to recruit 48 care homes in each participating country, collecting data on 192 deceased residents, from care home staff members, general practitioners (GPs)/physicians and relatives of the resident, collecting data on patient and family palliative care outcomes [11–15].

**Fig. 1:**
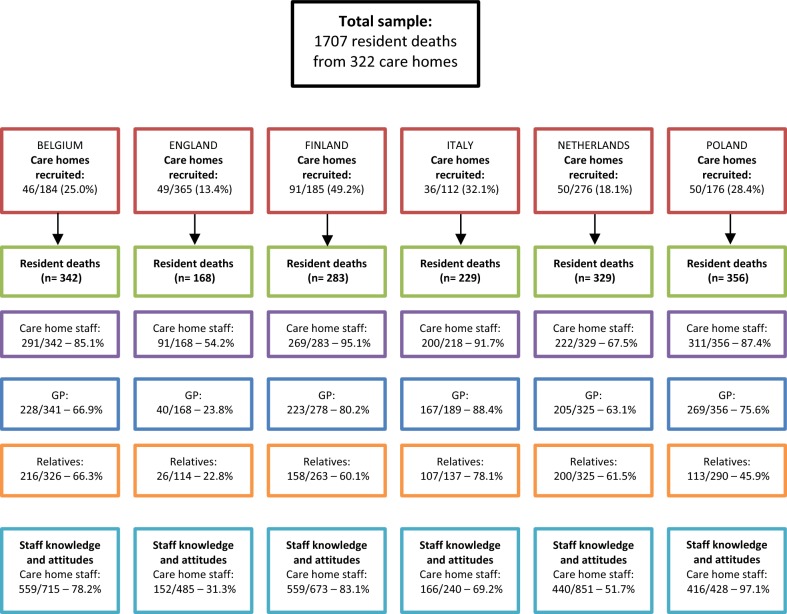
Recruitment and response rates, by country, in the PACE study

Figure 1 displays the recruitment and response rates for each questionnaire, for each participant per country. The response rates for care homes in England were lower than in the other countries involved in the study. This paper aims to describe the challenges encountered in conducting the study in England to inform the design and conduct of future international research in care homes. It will specifically explore the challenges encountered in developing and piloting the study, creating a sampling framework, contacting and recruiting care homes, conducting research visits and increasing response rates.

## Main text

### PACE study development and piloting

During the PACE study development, focus was on ensuring questionnaire data collected across countries would be comparable. Country specific questions and terminology were included, where appropriate, to reflect variation in the funding and types of care homes available. In each country, study documentation was piloted to ensure wording and formatting were accessible; in England this included feedback from a public involvement group, staff from two care homes and three GPs. Feedback centred on whether questionnaire respondents were required to provide written informed consent for their answers to be used before returning the questionnaire. It was agreed that return of the questionnaire would imply consent, providing that this was clearly stated in the participant information leaflet. A 3-month delay following the death on sending the relative questionnaire and signposting to bereavement services was also requested. This lag time extended the study cut-off date for returned data in England.

Two ethical issues were identified in study development, which potentially affected all countries involved in the study. The first issue concerned how care homes could provide confidential data on residents without breaking anonymity; to accommodate this the care home retained all resident identifiable data during the study and posted any questionnaires to recipients. A second ethical issue concerned whether relatives could be confused as to who would see their questionnaire responses, Lancaster University or the care home, which raised questions regarding confidentiality. Changes to the study process or documentation requested during the approvals process in England were often problematic as it reduced comparability with previously agreed documentation from the other countries in the study.

### Creating a sampling framework

**Fig. 2:**
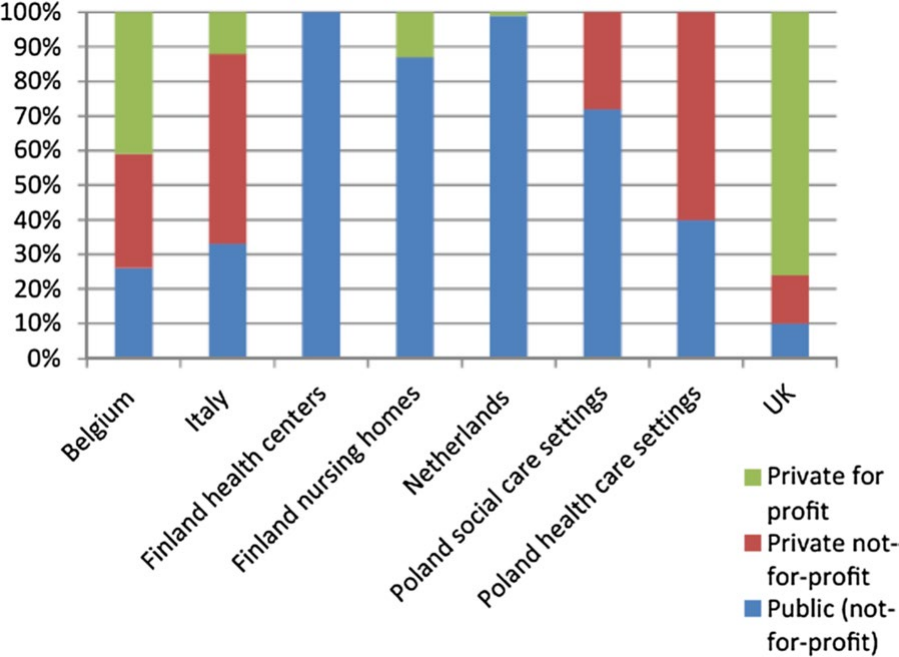
Care home providers by organizational status in each country involved in the PACE study [10]

To identify and recruit care homes, a stratified sample was created for each country based on care home region, type, size and organisational status, using national registers and based on estimated average deaths in each country over a 3 months period. In England, the data from the Care Quality Commission (CQC) was used, including the characteristics, contact details and reports on care quality from around 8000 care homes [16]. The problems encountered in England compared to the other countries in the study may reflect the variation in the long-term care economy across Europe—England has a significantly higher proportion of privately owned, for profit care homes (Fig. 2).

In England, the study excluded 396 local authority and NHS owned care homes as it would not have been possible to apply for local NHS approvals during the study period. In addition, care homes rated as at risk or providing inadequate care during their last CQC inspection were excluded to avoid adding extra work to care homes that were struggling. Quality of care was determined using the care homes most recent CQC inspection report. Forty-eight care homes were randomly selected which met the quota identified in the sampling framework.

Data in the CQC dataset was occasionally out of date. High levels of staff turnover meant that the contact details of managers were sometimes incorrect, and the numbers of beds had changed; care homes which were classed as small in the sampling framework were reclassified as large and no longer fitted into the sampling framework quota, and vice versa. The lag time of 3 to 6 months between CQC inspections the subsequent rating and report being published online meant that the research team were required to review CQC ratings on an ongoing basis.

### Contacting and recruiting care homes

Care homes identified in the sampling framework were contacted by post, with a follow up phone call from the research team 2 weeks later. E-mail contact led to substantially more responses than postal contact. Contacting care home managers by telephone was problematic, it took on average three phone calls to a care home before a manager or deputy manager could be reached. Care homes which were unresponsive after five phone calls were not followed up.

Within 3 months, it was clear that the current approach was unlikely to meet the recruitment target within the study period. The research team decided to advertise the study through the Enabling Research in Care Homes Programme (ENRICH) and in care home magazines [17]. The care homes involved in the ENRICH network were classed as ‘research ready’ and had indicated that they were interested in taking part in research. Nineteen care homes were recruited through the ENRICH network and advertising.

**Fig. 3:**
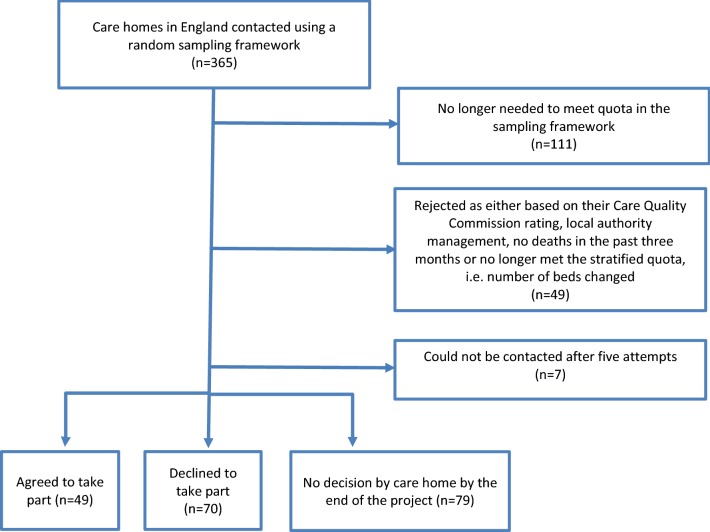
Recruitment of care homes in England, in the PACE study

Figure 3 shows the care home recruitment for England. Reasons for decline included being too busy, preparation for an upcoming CQC inspection, managers feeling uncomfortable providing information on a deceased resident and a view that palliative care was not part of the services provided by the care home. There were no statistically significant differences in terms of quality of care between care homes that agreed to take part in the study and those that declined, or between care homes identified through random sampling and those identified through the ENRICH network and advertising.

### Conducting research visits

Research visits to the recruited care homes were organised 2 months in advance and confirmed by post. At the research visit, it was common for care home managers to either have forgotten about the study or were not at the care home when the researcher arrived. A reminder call was made by a member of the research team 1 week before the visit to avoid this. As the study progressed, the importance of identifying specific times to visit care homes, avoiding medication rounds and GP visits, were recognised. As care homes are busy, unpredictable environments focused foremost on providing resident care, it was sometimes difficult to find a quiet, private room to discuss the study with the care home manager.

Accessing information on deceased care home residents was seldom straightforward. The data provided was sourced solely from the care home and relied on the quality of their record keeping. There is no linked computer system across care homes in England; how resident data is collected, updated and stored is locally determined. Some care homes used a paper-based system and care home administrators were asked to source information. Data could be stored in separated places; collated from CQC submissions, medical files and address books. Data on residents who had died in the past 3 months had occasionally been archived, either within or outside the care home. Depending on the number of deaths within the care home, the researcher visit could last up to 5 h.

It was anticipated that on average, there would be at least four deaths per care home over a 3-month period; in practice the number was lower. The average number of deaths across the care homes was three, in care home with nursing this was slightly higher, five deaths compared to two in care homes without nursing. As the visits were conducted between June and December, it is possible that seasonal variation in deaths could explain the discrepancy.

### Recruitment/response rates from care home staff, GPs and relatives

At the research visit, care home managers were asked to identify the staff member who was most involved in the resident’s care, which in practice was difficult to determine; a senior staff member was often asked to complete questionnaires on more than one deceased resident. Due to high staff turnover, the staff member closest involved in the residents care was sometimes no longer employed in the care home at the time of the research visit. Care home staff found it difficult to complete the questionnaire if a resident had recently been admitted to the facility, if the death had occurred in hospital or if they were asked to complete questionnaires on multiple residents.

In England, all GP care is provided externally to the care home and it is common for a care home to use only one GP practice; this led to GPs receiving questionnaires on multiple residents, potentially leading to questionnaire fatigue. The research team received uncompleted questionnaires from GPs who had only become responsible for the resident shortly before their death and did not feel qualified to complete a questionnaire on their care. Participants were not offered a monetary incentive to complete a questionnaire, and in some cases, GPs requested payment prior to questionnaire completion, which could not be provided.

Questionnaires for relatives of deceased residents were also sent out by post 3 months after the death. In some cases, either a relative could not be identified or it was thought by the care home manager to be inappropriate to contact a relative, i.e. if the relative was in poor health (n = 54).

Questionnaires on staff knowledge and attitudes to palliative care were only sent out to staff on duty at the time of the visit, therefore night and weekend staff may be underrepresented. One care home manager found it difficult to delimit staff members who were involved in care compared to those who were involved in domestic duties. In some care homes staff took on a number of roles depending on demand and all staff had training in care.

## Limitations

The extent to which the obstacles discussed in this paper are intrinsic to care home research, compared to the focus of the study, i.e. palliative care is unclear. In the PACE study, support from the care home manager, enthusiasm among staff and identifying a dependable contact person were imperative in increasing response rates. A major barrier to engagement was that a single research visit to a care home with little prior contact did not allow a relationship with the research team to develop. Initiatives such as ENRICH can enable care home involvement in research; however whether research ready care homes are representative of others nationally is uncertain [17, 18]. The study did not provide any incentives or reimbursements for care home staff, GPs and relatives to take part in the study, which may also explain the low response rate.

The experience of England in the PACE study demonstrates how conducting international studies within the legal, cultural and social norms of each country is challenging. Further research should explore the methodological challenges in this field. Open discussion of these challenges could inform the feasibility and development of research, especially in complex and sensitive areas such as palliative care.

## Data Availability

Not applicable.

## Abbreviations

CQC: Care Quality Commission
ENRICH: National Institute for Health Research: Enabling Research in Care Homes Programme
GP: general practitioner
MDS: Minimum Data Set
NHS: National Health Service
PACE: Palliative Care for Older People in care and nursing homes in Europe
